# Behavioral and emotional co-modulation during dog–owner interaction measured by heart rate variability and activity

**DOI:** 10.1038/s41598-024-76831-x

**Published:** 2024-10-24

**Authors:** Aija Koskela, Heini Törnqvist, Sanni Somppi, Katriina Tiira, Virpi-Liisa Kykyri, Laura Hänninen, Jan Kujala, Miho Nagasawa, Takefumi Kikusui, Miiamaaria V. Kujala

**Affiliations:** 1 Department of Psychology, Faculty of Education and Psychology, University of Jyväskylä, PO BOX 35, 40014 Jyväskylä, Finland; 2 Faculty of Veterinary Medicine, University of Helsinki, PO BOX 66, 00014 Helsinki, Finland; 3 Department of Animal Science and Biotechnology, Azabu University, 1-17-71 Fuchinobe, Chuo-Ku, Sagamihara, Kanagawa 252-5201 Japan

**Keywords:** Canine, Electrocardiography, Synchrony, Emotion, Dog–human interaction, HRV, Activity, Physiology, Psychology

## Abstract

Behavioral and physiological synchrony facilitate emotional closeness in attachment relationships. The aim of this pseudorandomized cross-over study was to investigate the emotional and physiological link, designated as co-modulation, between dogs and their owners. We measured the heart rate variability (HRV) and physical activity of dogs belonging to co-operative breeds (n = 29) and their owners during resting baselines and positive interaction tasks (Stroking, Training, Sniffing, Playing) and collected survey data on owner temperament and dog–owner relationship. Although overall HRV and activity correlated between dogs and their owners across tasks, task-specific analyses showed that HRV of dogs and owners correlated during free behaving (Pre- and Post-Baseline), whereas the activity of dogs and owners correlated during predefined interaction tasks (Stroking and Playing). Dog overall HRV was the only predictive factor for owner overall HRV, while dog height, ownership duration, owner negative affectivity, and dog–owner interaction scale predicted dog overall HRV. Thus, the characteristics of dog, owner, and the relationship modified the HRV responses in dog–owner dyads. The physiology and behavior of dogs belonging to co-operative breeds and their owners were therefore co-modulated, demonstrating physiological and emotional connection comparable to those found in attachment relationships between humans.

## Introduction

Behavioral and physiological synchrony facilitate communication between human individuals by enhancing reciprocity, closeness, and trust through the experience of shared emotional states^1,2^. In the mother-infant interaction, simultaneous synchrony at the behavioral, neural, and physiological level appears to be a prerequisite for a human infant’s normal social development and ability to form secure attachment bonds later in life^3^. The co-evolution of dogs *(Canis familiaris)* and humans^4^ has resulted in close cooperation and mutual attachment bonds between members of the two species^5,6^, resembling in certain aspects the bond between mothers and children. In particular, dogs exhibit attachment behavior towards their owners^7,8^ and the attachment bond is physiologically regulated by the oxytocin loop between the dog and owner^9^. Furthermore, dog behavior may be similarly synchronized with humans as with individuals from the same species^10^, and the synchronization seems to be more evident when the social bond between the dog and the human is stronger^11^. Dogs also prefer the company of humans who exhibit more behavioral synchrony with them^12^. However, despite the worldwide popularity of the dog as a beloved family member, little is known about the emotional and physiological mechanisms modulating the relationship between dogs and their owners.

Although behavioral and hormonal synchronization between dogs and humans has recently received increased scientific attention^10,13^, the synchrony of the autonomic nervous system (ANS) within dog–owner dyads remains relatively unexplored. ANS regulates involuntary bodily functions via two of its branches, namely the sympathetic nervous system (controlling physiological arousal and alertness) and the parasympathetic nervous system (regulating digestion and rest)^14^. ANS synchrony is a well-studied form of physiological synchrony in humans; the phenomenon has been observed in all attachment relationships from parent-infant dyads to romantic couples and friends^15^. Changes in ANS can be assessed from heart rate variability (HRV), which represents the time intervals between adjacent heart beats^16^. HRV reflects the balance between the parasympathetic and sympathetic nervous system, with high values indicating low arousal states and low values indicating high arousal states in general. HRV is affected by several physiological and psychological factors^17^, such as physical activity and emotions. However, in emotion-related HRV studies on freely moving animals, the physical activity is often overlooked even though it directly modifies cardiac function^18^ and usually influences the validity of cardiac recordings^19^.

The dog–human interaction impacts humans on a physiological level; interaction with a dog increases HRV and oxytocin levels and decreases cortisol levels of humans^20,21^. For example, in elderly foster dog caregivers who place high value on the company and emotional support of their foster dog, the mere presence of the dog throughout the day increased the caregiver’s HRV^22^. The dog–owner relationship also affects the dog’s physiological responses. The dog owner’s higher emotional closeness with the dog is associated with higher HRV in dogs during short-term interaction^23^. Furthermore, the owner’s higher level of attachment towards the dog is related to lower cortisol levels in dogs^24^. Thus, analogously to human parents, dog owners may have a calming effect on their dog, probably because the strong emotional bond provides feelings of safety and buffers the dog from high-level stress^25^. According to previous findings, the owner’s characteristics influence the dog–owner relationship. For example, the owner’s higher negative affectivity and orienting sensitivity are related to stronger emotional closeness with the dog^26^. In fact, owner-related factors appear to affect the relationship even more than the characteristics of the dog^13,27,28^.

Although dogs in general have a species-typical predisposition for affiliation with humans^29^, their sensitivity for human behavior differs between breed types^26,30^. Dog breeds bred for close cooperation with humans seem to be more sensitive to human behavior and emotions than more independent breeds. For example, herding dogs are highly responsive to human cues^31^ and their cortisol levels correlate with those of their owners over a time course of a year^13^, whereas similar synchronization is not apparent in ancient breeds or solitary hunting breeds^32^. The personality or temperament of the owner seems to have stronger influence on herding and solitary hunting dogs compared with ancient breeds^26,32^. In addition to breed type, the time lived together may modify the physiological and emotional connection between dogs and owners. Dog–owner dyads that have lived together longer show enhanced emotional contagion^33^ and long-term owners report stronger attachment to their dog^34,35^, suggesting mutual bonding taking place over time.

The definition of synchrony and the various methods used in measuring it (i.e. Pearson correlation, cross-correlation, and times series analysis) in the literature are rather disorganized, leading to conflicting results and complicating a deeper understanding of the phenomenon^15^. In this study, our main aim was to characterize emotion-related behavioral and physiological synchrony between dogs and their owners during different tasks involving interaction and resting baselines and to examine demographic, owner temperament and dog–owner relationship factors affecting the synchronization. We studied the physiological synchrony in a broader sense, with correlation of averaged HRV over several minutes, instead of moment-to-moment changes in HRV between the dog and owner. Thus, to clarify the conceptual field, we will use the term “co-modulation” instead of synchrony to refer to the simultaneous modifying effects of dogs on the owners and vice versa within a larger timescale.

The main objectives of this study were to examine (1) whether the HRV and activity of dogs and their owners are co-modulated during social interaction; (2) whether different kinds of social interaction lead to differing HRV and activity co-modulation between dogs and their owners; and (3) which demographic, relationship, or temperament factors explain the overall HRV of the dogs and the owners across the study. We focused on co-operative breeds, which appear to be the most suitable candidates for modulating their behavior and physiology in response to their owners^13,26^.

## Materials and methods

### Ethics statement

The study had prior approval by the ethical board of the University of Jyväskylä (26.6.2020; statement #760/13.00.04.00/2020; amendment 05/2022). All dog owners gave written informed consent before participation in the study. Experimentation was performed in accordance with the relevant guidelines and regulations of the Finnish National Board on Research Integrity (https://tenk.fi/en). The study is reported in accordance with ARRIVE guidelines (https://arriveguidelines.org).

### Subjects

A total of 30 healthy pet dogs from 13 breeds (from the categories of FCI1, Sheepdogs and Cattledogs and FCI8, Retrievers, Flushing Dogs, and Water Dogs; see Supplementary Table 1) participated in the study with their owners. Subjects were recruited with targeted social media postings (Facebook) and through the smartDOG company (Riihimäki, Finland, https://www.smartdog.fi/english/), which offers cognitive testing for dog owners. Exclusion criteria for the dogs were aggressiveness or fearfulness towards unfamiliar people, estrus in female dogs, and acute disease or medication affecting cardiac functions. Only medium-sized dogs (10–30 kg) were included to minimize confounding factors related to HRV values due to large variation in body size^36^. Exclusion criteria for the owners were age < 18 years or acute illness or related medication.

Altogether five dog–owner dyads were excluded from the analyses due to following: owners’ abnormally low baseline HRV (n = 2)^37^, owners’ body mass index > 40 kg/m^2^ (n = 2)^38^, and missing dog electrocardiogram (ECG) data (n = 1). Thus, the final sample consisted of 25 dog–owner dyads. Twelve male and 13 female dogs were included. Mean ± SD age was 5.9 ± 2.6 years and weight was 20.4 ± 4.3 kg. The dog owners (23 females and 2 other or did not respond) were 40.8 ± 8.1 years of age and weighed 71.3 ± 13.5 kg. All dogs lived indoors with the owners and 23 of them were acquired as puppies (age < 12 weeks). Majority of the dogs (n = 17) had been actively trained for a dog sport (e.g. agility, obedience training), and one dog had been trained for hunting.

### Questionnaires of dog–owner relationship and owner temperament

Before the interaction study, dog owners answered web-based questionnaires to collect general demographic information, temperament with the Adult Temperament Questionnaire (ATQ-R)^39^, and dog–owner relationship with the Monash Dog–owner Relationship Scale (MDORS)^40^. The adult temperament questionnaire consists of the following four factors: *negative affectivity* (sampling the subject’s tendency to experience negative thoughts or emotions), *effortful control* (sampling the ability of the subject to control one’s actions utilizing cognitive reappraisal), *extroversion* (sampling the subject’s tendency to experience positive emotions and to exhibit sociability), and *orienting sensitivity* (sampling the tendency of the subject to pay attention to small details in the environment). The dog–owner relationship was assessed with following factors: *Emotional Closeness* (MDORS-EC), *Perceived costs* (MDORS-PC), and *Dog–owner Interaction* (MDORS-DOI). The reliability of questionnaire data was checked by calculating Cronbach’s α (Cr-α) for each factor of both questionnaires (Table 1).

**Table 1:** Average scores of the ATQ-R and MDORS factors (scale 1–5) and reliability values (Cr-α) of factors.

Questionnaire	Factors	Mean ± SD	Range	Cr-α
ATQ-R	Negative affectivity	4.12 ± 0.98	2.53–6.00	0.77
ATQ-R	Effortful control	4.53 ± 0.63	3.27–5.87	0.75
ATQ-R	Extroversion	4.29 ± 1.10	2.13–5.93	0.63
ATQ-R	Orienting sensitivity	4.69 ± 0.52	3.80–5.93	0.82
MDORS-EC	Emotional closeness	3.98 ± 0.69	2.43–4.80	0.84
MDORS-PC	Perceived costs	1.67 ± 0.32	1.10–2.13	0.89
MDORS-DOI	Dog–owner Interaction	3.61 ± 1.17	1.97–4.97	0.62

### Experimental procedures

The study followed a pseudorandomized cross-over design, with a correlational quantitative approach utilized regarding the relationship between the background factors and measured variables. The experiments were conducted in the Faculty of Education and Psychology, University of Jyväskylä in June 2022. The test room with two windows (approximately 30 m^2^) was furnished with eight beanbag chairs (one in the middle and others against walls), cabins, a table, and a sink. The floor was covered by wall-to-wall carpet and two large canvas screens were placed in front of the windows to cover the view outside. Two web cameras were attached to the walls opposite one another, and one video camera was located on top of a closet. The owners were asked to bring along treats and a few favorite toys of their dog. There were also treats, a sniffing mat, toys, and a water bowl in the test room reserved for the dogs.

Before the experiment, the dogs became accustomed to the premises for approximately 30 min, roaming freely in the experiment room, while the owners sat down and received instructions from the researchers. ECG devices (Bittium Faros 180 TM, Bittium Biosignals Ltd, Kuopio, Finland) were then first attached to the dog and then to the owner, allowing more time for the dog to adjust to the sensors. During the test (approximately 53 min in total duration), the dog and the owner were alone in the room while the researchers observed them from another room via video and audio connection; in case the owner needed additional advice, an audio connection was opened bidirectionally by the experimenters. Throughout the study, written instructions were shown on a computer screen as the tasks changed.

The experiment started and ended with a resting baseline period, with four different tasks in between (Table 2). The different interaction tasks were selected to model natural dog behavior and dog–owner interaction with distinct levels of physical activation (e.g. Playing vs. Stroking). Between every task, there was a 3-min break, during which owners were instructed to take a break and sit down. Based on prior studies that utilized 1-min washout periods^41,42^, 3-min washout periods were considered sufficient to mitigate the influence of preceding tasks. Tasks were conducted in fixed order. There were two different orders to diminish the possible order effect but to retain balance between active and calm phases. Playing was always the last task (before Post-Baseline) to minimize loss of data in case the ECG devices loosened during the play. Thus, 14 dog–owner dyads followed the task sequence of Pre-Baseline, Stroking, Training, Sniffing, Playing and Post-Baseline, and 11 dyads followed the task sequence of Pre-Baseline, Sniffing, Training, Stroking, Playing and Post-Baseline.

**Table 2:** Detailed description of experimental tasks.

Tasks	Duration (min)	Expected emotional valence	Expected activity level	Description
Pre-baseline	10	Neutral	Low	The owner was instructed to sit down and relax, and act normally towards the dog. The dog was allowed to behave freely
Stroking	6	Positive	Low	The owner stroked the dog without restricting it from moving and only if the dog was willing to be stroked
Training	6	Positive	High	The owner trained the dog with positive reinforcement (food rewards). The owner was asked to avoid commands, including lying down and rolling over, to protect the ECG devices
Sniffing	6	Positive	Low	The owner took a sniffing mat, with hidden treats in it, from a closet and gave it to the dog for sniffing. In case the dog found all the treats before the task was finished, the owner added extra treats into the mat
Playing	6	Positive	High	The owner and the dog played together either with familiar toys (taken from home) or with new toys (provided by the researchers)
Post-baseline	10	Neutral	Low	The owner was instructed to sit down and relax and act normally towards the dog. The dog was allowed to behave freely

### Cardiac acquisition

**Fig. 1:**
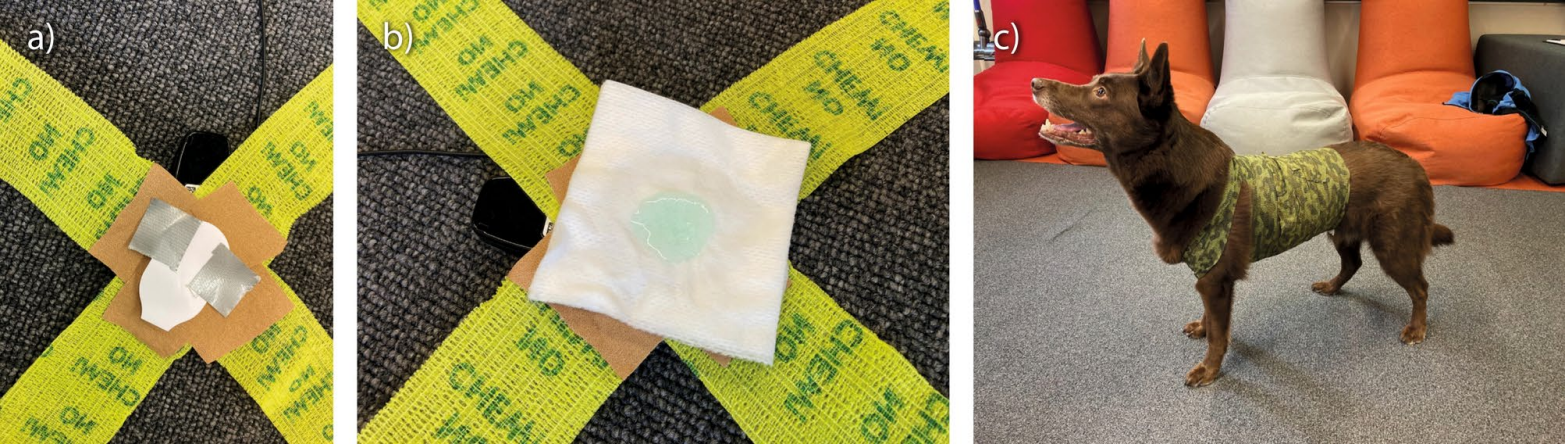
(**a**) Preparation phase: electrodes were attached to self-adhesive elastic bandage and protected with cover and adhesive tape. (**b**) To optimize the connection, saline solution and conductive ultrasound transmission gel (AquaSonic100, Parker Laboratories Inc., Fairfield, CA, USA) were applied to the cotton sheet with electrode location. (**c**) To keep the electrodes firmly in contact with the skin, a self-adhesive elastic bandage was applied carefully around the dog chest on top of the electrodes.

ECG signals measured at a sampling rate of 1000 Hz were acquired from the dogs and the owners using Bittium Faros 180 TM devices (Bittium Biosignals Ltd, Kuopio, Finland). The devices worn on the dog and the owner were synchronized with the eMotion Faros Manager 2.3.0 software prior the experiment. The procedure of attaching electrodes to dogs was conducted similarly as Katayama et al.^33^. The dogs’ fur was combed out of the electrode locations and the skin was cleaned with alcohol. One ECG electrode (Kendall™ hydrogel foam H92SG electrodes, 57 × 34 mm) was attached on the area of the manubrium (i.e. dog’s forechest) while the other electrode was attached on the area of the xiphisternum (i.e. dog’s chest) (Fig. 1). Similar electrodes were used for humans. The electrode attachment was conducted in a standardized manner; the skin was cleaned with alcohol and the electrodes were attached to the chest and close to the armpit with the electrode pads.

### Cardiac data preprocessing

Human ECG data were corrected for artefacts with Kubios Scientific 4.0.3 software (Kubios Oy, Kuopio, Finland). Due to better accuracy^43^, automatic correction, in which artefacts are detected from a time series consisting of differences between successive RR intervals, was selected instead of different correction thresholds.

HRV in dogs functions somewhat distinctively compared with humans in part due to the prominent respiratory sinus arrhythmia in dogs^44^. Consequently, the analysis of dog HRV is laborious with algorithms developed for human ECG data. We collaborated with Kubios researchers to develop a beta version of the software suitable for canine HRV analysis, which was used in the dog cardiac data preprocessing with automatic correction adjustment. The resulting protocols for canine HRV analysis was released for commercial use in Kubios Scientific software version 4.0.3 (https://www.kubios.com/release-notes/).

Thereafter, valid ECG segments of each task (epoch of 1–3 min fulfilling the criterion of ≤ 5% corrected beats per segment) were selected for the analysis. In the selection of ECG segments from each task, fully overlapping time intervals were used in both owner and dog segments, except three segments were only partially overlapping (see Supplementary Table 2). From valid ECG segments, average root mean square of successive differences between normal heart beats (RMSSD) was extracted by Kubios software. Finally, overall RMSSD value for each dog and owner was calculated by averaging these task-specific RMSSD values across all tasks. RMSSD was used as a measure of HRV as it is less sensitive to motion compared with other HRV measures^19,23^.

### Activity measurement

3D acceleration signals measured at a sampling rate of 100 Hz were acquired from the dogs and the owners using the same Bittium Faros 180 TM devices that were used to record the ECG signals. The physical activity value at each timepoint was determined as the square root of the squared sum of the acceleration in the three orthogonal directions (x, y, z). Before computing the squared sum, the acceleration values were subjected to first-order differencing (differences between successive time-points) to account for the scaling difference in the acceleration values across individuals. These values were then averaged within the same time intervals as for the HRV calculation. Task-specific activity values were averaged to compute the overall activity value for each dog and owner. Absolute value of accelerometer vector |v| was used as an activity measure.

### Statistical analysis

The normality of overall and task-specific RMSSD and activity data were tested with Shapiro–Wilk tests, confirming non-normal distribution in part of the RMSSD data and in part of the activity data. For conformity in the results, the correlation of dog and owner RMSSD and dog and owner activity were analyzed with Spearman correlation (*ρ*) with statistical analysis software SPSS 28.0 (IBM, New York, NY, USA). Further, to test for possible pseudocorrelation between dog and owner RMSSD, data of dogs and owners were randomly assigned to new dog-(non)owner dyads and the correlation between overall RMSSD in the new dyads was analyzed with Spearman correlation. For the correlation figures, all RMSSD and activity values were z-scored due to large scaling differences between the values of dogs and owners. A z*-*score or standard score describes how far a data point is from the mean using standard deviations. This can be negative or positive depending on whether the observation is above or below the mean.

The general HRV and activity differences between the tasks in owners and dogs were analyzed with Friedman’s two-way ANOVA.

To predict independent variables affecting the owner overall RMSSD, multivariate linear regression with stepwise method was applied. The list of independent variables included o*wner’s age, owner’s weight, owner’s BMI, activity of owner, activity of dog, dog’s overall RMSSD, dog’s sex, dog’s age, ownership duration, years in practicing dog sports, owner’s temperament* (*negative affectivity, extroversion, effortful control, and orienting sensitivity*), and *MDORS factors* (*emotional closeness, perceived costs, and dog–owner interaction*). The inclusion criterion was set to *p* < 0.05 and exclusion criterion to *p* > 0.10. The final model included only statistically significant variables. The assumptions of multivariate linear regression were satisfied.

Likewise, multivariate linear regression with stepwise method was applied to predict independent variables affecting the dog overall RMSSD; inclusion criterion was set to *p* < 0.05 and exclusion criterion to *p* > 0.10. The list of independent variables included *dog’s sex, dog’s weight, dog’s height, dog’s age, activity of dog, activity of owner, owner’s overall RMSSD, ownership duration, years in practicing dog sports, owner’s temperament (negative affectivity, extroversion, effortful control, and orienting sensitivity)*, and *MDORS factors (emotional closeness, perceived costs, and dog–owner interaction)*. The final model included only statistically significant variables. The assumptions of multivariate linear regression were satisfied.

The correlation between the study variables of dog–owner relationship, owner temperament, dog and owner demographics, and dog and owner overall HRV and activity were analyzed with Spearman correlation.

## Results

**Fig. 2:**
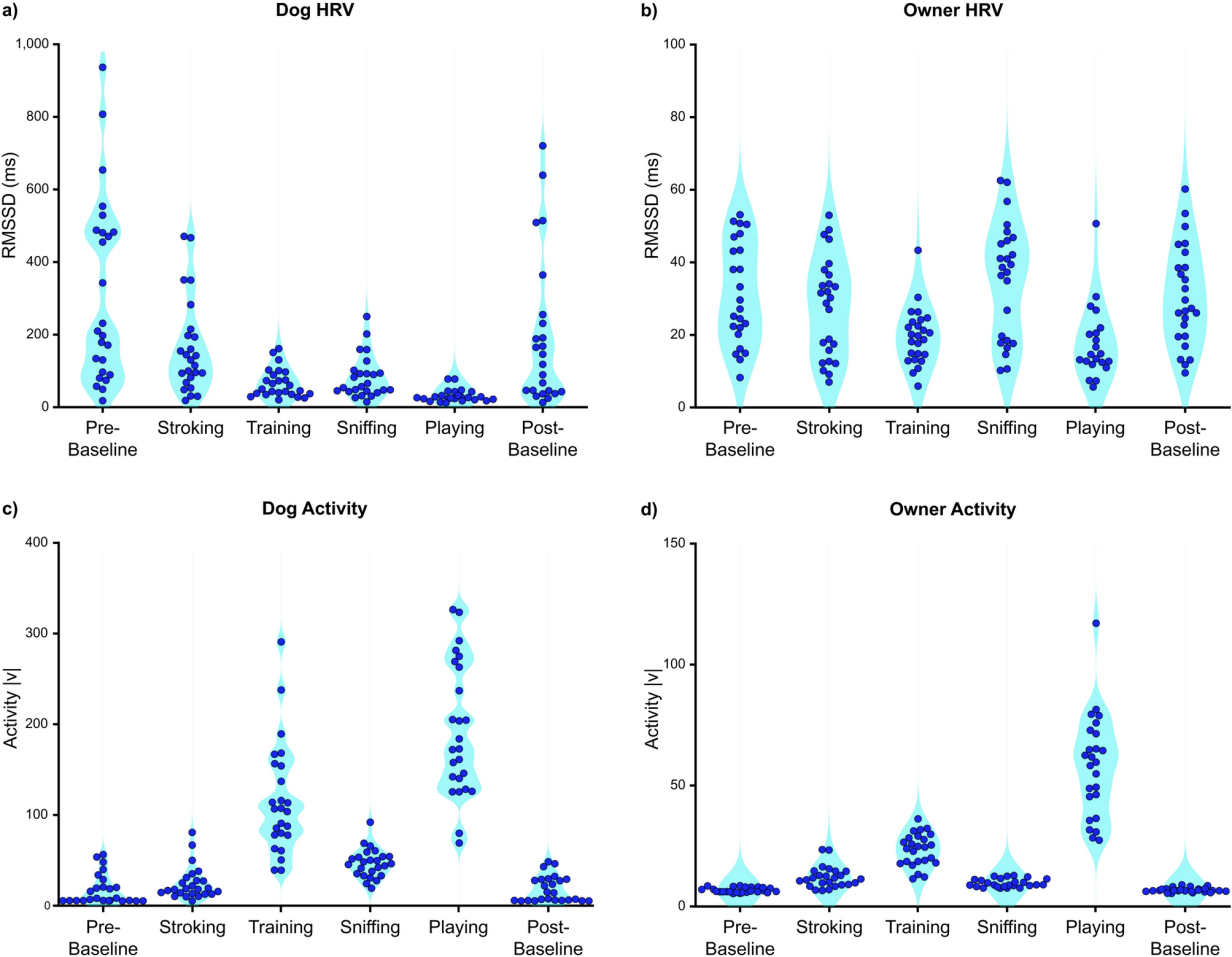
Descriptive RMSSD and activity scores of each dog (**a,c**) and each owner (**b,d**) during six tasks (Pre-Baseline, Stroking, Training, Sniffing, Playing, Post-Baseline). Each dot represents one individual. The same time intervals (epoch of 1–3 min) were used in calculating average task-specific RMSSD and activity scores in both dogs and owners to enable the time-locked comparison. |v| = absolute value of accelerometer vector.

The overall (mean ± SD) RMSSD values of dogs and owners were 145 ± 97 ms and 27 ± 11 ms, respectively. The overall activity of dogs and owners were 69 ± 27 |v| and 19 ± 4 |v|, respectively. The median and IQR for dogs were 136 ± 142 ms (RMSSD) and 67 ± 40 |v| (activity), and for owners 26 ± 21 ms (RMSSD) and 20 ± 6 |v| (activity). Generally, dog RMSSD values were approximately tenfold higher than the owners, particularly during baselines (Fig. 2a,b). Some of the dogs were calm and sedentary during Pre- and Post-Baseline, while others were more aroused and active according to the task-specific HRV and activity data (Fig. 2c). The activity of the owners was generally low during baselines (Fig. 2d) and HRV varied considerably, reflecting high individual variability. The average HRV of the owners was lowest during Playing and Training (HRV comparisons: Playing *vs.* other tasks and Training *vs*. other tasks, all *p* < 0.010, except Training vs. Playing *p* = 0.612; Supplementary Tables 3, 4). The average activity of owners was highest during Playing and Training (Activity comparisons: Playing *vs.* other tasks and Training *vs.* other tasks, all *p* < 0.010, except Training *vs.* Playing *p* = 0.070; Supplementary Tables 3, 5). The average HRV of dogs was lowest during Playing (HRV comparisons: Playing *vs.* other tasks, all *p* < 0.010; Supplementary Tables 3, 6). The average activity of dogs was highest during Playing and Training (Activity comparisons: Playing *vs.* other tasks and Training *vs.* other tasks, all *p* < 0.020, except Training *vs.* Playing, *p* = 0.059; Supplementary Tables 3, 7).

### Correlation between dog and owner heart rate variability

**Fig. 3:**
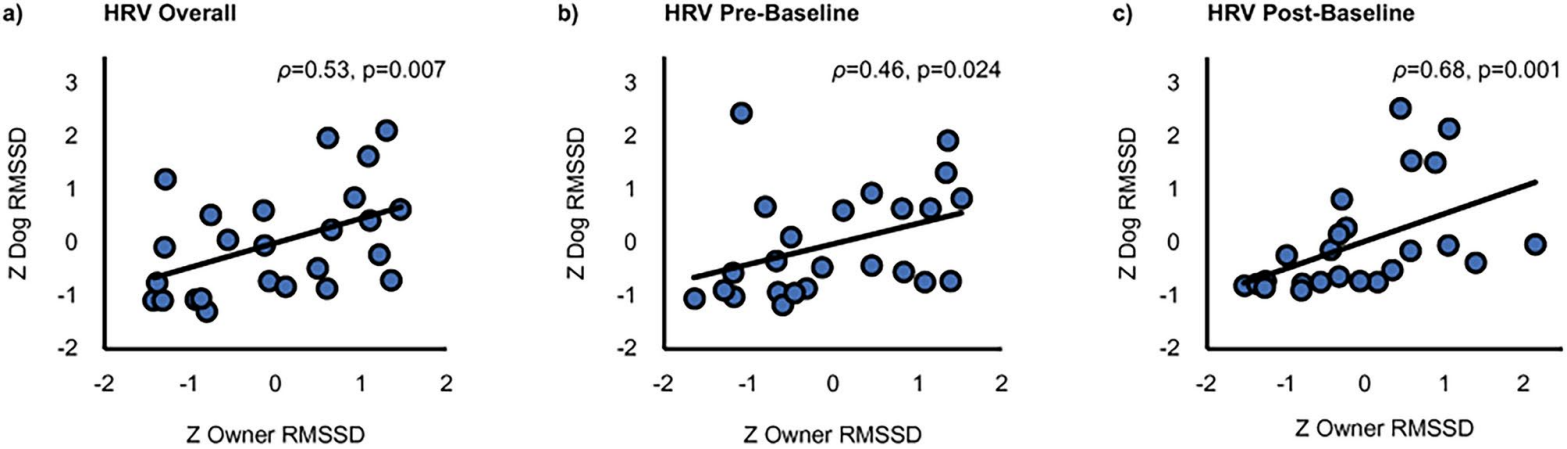
RMSSD correlation between dogs and owners (**a**) across the tasks, (**b**) during Pre-Baseline, and (**c**) during Post-Baseline. All the RMSSD values are z-scored.

The overall RMSSD of dogs and their owners across all tasks correlated statistically significantly (*ρ* = 0.53,* p* = 0.007, bias-corrected and accelerated 95% confidence intervals [BCa 95% CI] 0.173–0.753; Fig. 3a). In addition, a statistically significant positive correlation of dog and owner RMSSD was found during Pre-Baseline (*ρ* = 0.46, *p* = 0.024, BCa 95% CI 0.194–0.762; Fig. 3b) and Post-Baseline (*ρ* = 0.68, *p* < 0.001, BCa 95% CI 0.434–0.827; Fig. 3c). A marginally significant positive correlation was found during Stroking (*ρ* = 0.34, *p* = 0.097, BCa 95% CI − 0.139–0.691) and Playing (*ρ* = 0.41, *p* = 0.070, BCa 95% CI 0.037–0.682).

### Correlation between dog and owner activity

**Fig. 4:**
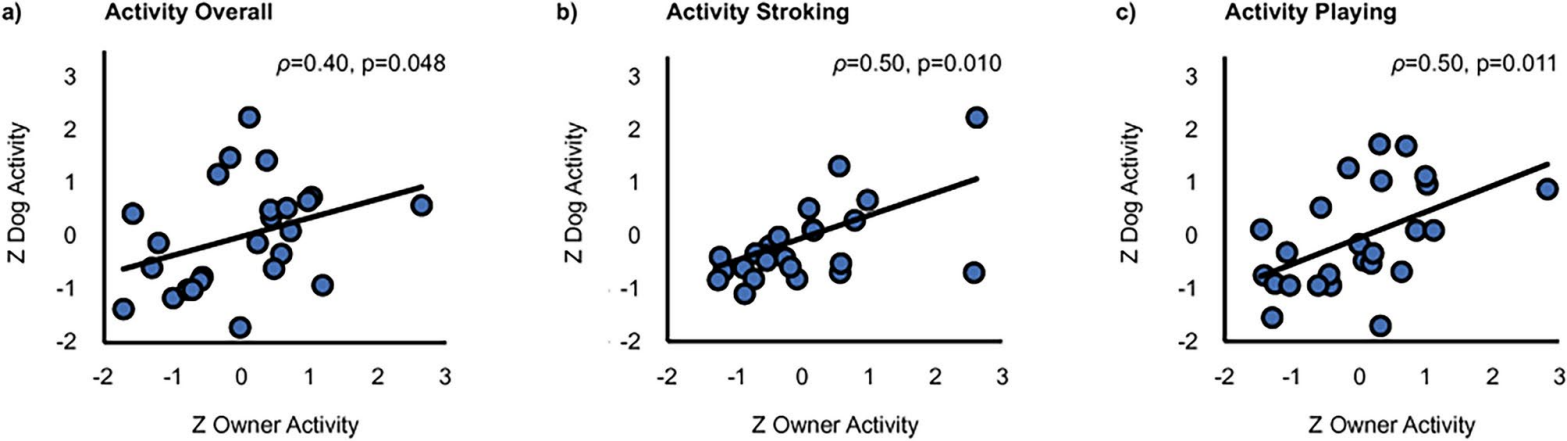
Activity correlation between dogs and owners (**a**) across the tasks, (**b**) during Stroking, and (**c**) during Playing. All activity values are z-scored.

The overall activity across all tasks correlated statistically significantly between dogs and their owners (*ρ* = 0.40, *p* = 0.048, BCa 95% CI 0.053–0.653; Fig. 4a). In addition, a statistically significant positive correlation of dog and owner activity was found during Stroking (*ρ* = 0.50, *p* = 0.010, BCa 95% CI 0.048–0.824; Fig. 4b) and Playing (*ρ* = 0.50, *p* = 0.011, BCa 95% CI 0.148–0.744; Fig. 4c). A marginally significant positive correlation was also present during Post-Baseline (*ρ* = 0.35, *p *= 0.083, BCa 95% CI − 0.088–0.650).

### Correlation between dog and non-owner heart rate variability over the tasks

The overall RMSSD of dogs and the overall RMSSD of randomized owners were not correlated statistically significantly (*ρ* =  − 0.018, *p* = 0.930).

### Predictive variables of dog and owner overall RMSSD

Multivariate linear regression analysis of *owner’s overall RMSSD* (r^2^ = 0.212, Cohen’s f^2^ = 0.27) showed that higher RMSSD in dogs was associated with higher RMSSD in owners (*dog overall RMSSD,* β = 0.051, 95% CI 0.008–0.095, *p* = 0.024).

Multivariate linear regression analysis of *dog’s overall RMSSD* (r^2^ = 0.67, Cohens’s f^2^ = 2.02) showed that *dog’s height, ownership duration, owner’s negative affectivity,* and *Dog–owner Interaction* (MDORS-DOI) statistically significantly predicted the *dog’s overall RMSSD* (Table 3). Therefore, larger dogs and dogs of owners scoring higher in negative affectivity had higher RMSSD values. Instead, dogs who had been living longer with the same owner and shared more activities with their owners had lower RMSSD values.

**Table 3:** Statistical results of multivariate linear regression analysis predicting overall RMSSD of dogs.

Independent variable	Standardized β*	95% CI	p-value
Dog’s height (cm)	0.486	2.98 to 11.17	0.002**
Ownership duration (y)	− 0.436	− 25.09 to − 5.59	0.004**
Owner’s negative affectivity	0.361	13.90 to 96.66	0.011*
MDORS-DOI	− 0.316	− 114.30 to − 8.19	0.026*

*MDORS-DOI* Monash dog–owner relationship Scale: Dog–owner Interaction, β* regression coefficient standardized for other variables included in the model, *CI* confidence interval.

*p < 0.05; **p < 0.01.

### Correlations between study variables

*Emotional closeness* (MDORS-EC) correlated statistically significantly with *Dog–owner Interaction* (MDORS-DOI) (*ρ* = 0.507, *p* < 0.05) and *Ownership duration* (*ρ* = 0.512, *p* < 0.01). *Ownership duration* correlated statistically significantly with *Activity of dog* (*ρ* = 0.397, *p* < 0.05), *HRV of dog* (*ρ* =  − 0.569, *p* < 0.01) and *Age of dog* (*ρ* = 0.779, *p* < 0.001). *HRV of dog* correlated statistically significantly with *Activity of dog* (*ρ* =  − 0.525, *p* < 0.01) and *Height of dog* (*ρ* = 0.568, *p* < 0.01). See Supplementary Table 8 for all correlations between study variables.

## Discussion

In this pioneering work, we investigated emotion-related autonomic nervous system reactions as changes in HRV in both dogs and their owners during several positive interaction tasks and resting baselines while simultaneously tracking the activity of the participants. The main aim of this study was to examine the emotional and physiological co-modulation between dog and owner during interaction and to identify the factors that modify it. We measured HRV and activity data from the dogs and their owners across the study and collected detailed information on the subjects by several questionnaires. Our results show co-modulations in both HRV and activity levels during dog–owner interaction. However, the effects of HRV and activity were partially independent of each other. Specifically, the HRV of dogs and owners were correlated during times of free interaction, whereas the activity of dogs and owners were coordinated during predefined interaction tasks. These findings suggest emotional co-regulation of the HRV of dogs and owners beyond their correlations due to synchronized physical activity. The results also show that dog, owner, and their relationship characteristics predicted the overall level of arousal of dogs and owners.

### Co-modulation of HRV and physical activity

HRV and activity of dogs and owners correlated positively across all tasks, indicating that owners with high arousal levels had dogs with high arousal levels and the owner’s higher physical activity was related to the dog’s higher activity throughout the study. Generally, these results are consistent with previous findings, which demonstrated synchronization between dogs and their owners in behavior^10^ and hormonal levels^9,13^. As behavior often includes motion, the overall activity correlation reflects behavioral co-modulation in a broader sense. Physical activity has direct effects on metabolism and consequently on HRV^19^, thus it could be assumed that the HRV correlation between the dogs and owners may be a consequence of co-modulation in activity. However, neither dog nor owner activity explained dog or owner HRV in our regression models. Additionally, dogs and owners have a strong correlation^45^ in HRV, whereas their activity levels show a medium correlation, suggesting that other factors also modify the HRV correlation in addition to the activity.

In humans, emotional synchronization between closely attached individuals is reflected in HRV levels during social interaction^46,47^. Emotions influence the autonomic nervous system and hence HRV but instead of distinctive emotion-specific fingerprints, changes in HRV better reflect the general state of emotional arousal^48^. Emotional states of both high and low arousal levels appear shared from mothers to infants during interaction, whereas physical contact enhances the emotional contagion during high arousal states^49^. Interestingly, the arousal levels of dogs and their owners seem to reflect one another across a day when spending time in the same room irrespective of direct interaction^22^. Our results suggest that this physiological linking related to emotional arousal may also occur between dogs and owners during short-time interactions.

Nevertheless, correlation between two factors, such as the HRV of dogs and their owners, can also derive from identical external conditions, resulting in pseudocorrelation^50^. This kind of pseudocorrelation means that the individuals are merely similarly tuned to the outside factors, without directly affecting each other. Here, we examined the possibility of pseudocorrelation by randomizing the data of dogs and owners into new dyads, dogs with non-owners, to show that the HRV correlation existed only between dogs and their owners, not between dogs and random humans. This excludes the possibility that similar environmental factors, such as merely undergoing the same task, led to the HRV correlation. Considering the aforementioned points, the results suggest that part of the overall HRV correlation in the dog–owner dyads is accounted by emotional co-modulation between dogs and owners characteristics for attachment relationships^51^.

When elaborating the co-modulation of HRV and activity in dog–owner dyads within a more detailed, task-specific level, a significant correlation of HRV between dogs and their owners existed during Pre- and Post-Baselines. The activity of dogs and owners, instead, correlated significantly during Stroking and Playing. The results support the partially independent phenomena of activity and HRV co-modulation in the dog–owner interaction found across the tasks.

Physiological synchronization may occur during intentional projecting of the emotional state in other individuals^52^ and spontaneously without making an effort^15^. During the baseline periods of our study, when there were no external tasks, the dogs and owners could behave freely and concentrate more on each other, giving greater possibility for co-modulation of their arousal levels. In addition, the owners might have paid more attention to their dogs and initiated interaction more than usually because the focus of the research was openly disclosed to them. Furthermore, dogs generally mirror the owner’s reactions to novel situations and adapt their behavior according to the owner’s behavior^53^. In the beginning of the study, during Pre-Baseline, both dogs and owners might have been slightly stressed by the new situation and environment, thus increasing the dogs’ emotional referencing to the owners.

However, during Post-Baseline, the preceding task (Playing) and the correlation in activity during the task may have influenced the Post-Baseline HRV correlation together with the aforementioned factors. The dogs and owners had the highest average activity during playing and their activity levels were co-modulated. Typically, when playing with humans, dogs are interactive^54^, which explains the activity co-modulation between playmates in our study. As physical movement influences heart functions^19^, the delayed effects of the activity correlation during Playing as mutually increased arousal levels may partly account for the HRV correlation during Post-Baseline. Despite a 3-min washout period between consecutive tasks and a pseudo-randomized order of tasks, it is not possible to entirely eliminate order effects, as certain conditions were required to follow others, like Play and Post-Baseline. Furthermore, since playing strengthens the emotional bond between dogs and owners^55^, the playing task may have momentarily increased the emotional connection in dog–owner dyads, manifested as significant HRV co-modulation during Post-Baseline.

In contrast to HRV, the activity of dogs and owners correlated during Stroking. Despite the task remaining always the same, Stroking may have been pleasant or neutral for some of the dogs and unpleasant for others^41^, affecting the parameters of the study. According to expert observers’ subjective interpretations, the dogs who did not seem to enjoy stroking were restless and moved around considerably while the owners were trying to carry out the task. Instead, the dogs who liked to be stroked stayed still for the whole time and even initiated interaction with the owner if the owner stopped stroking the dog for a moment. Thus, the dog behavior was likely to affect the activity level of the owner during Stroking, resulting in a positive correlation of activity in the dyad. In previous research, controversial findings exist related to the positive outcomes of stroking^41^, demonstrating its challenging implementation in research settings and high context and individual dependency^56^. In the current study, most of the dogs were active in dog sports (e.g. agility or obedience training) and thus were used to participating in competitions; they might have been expecting a training context, anticipating action instead of calm interaction with the owner^57^.

During the Training and Sniffing in this study, neither the activity nor HRV of dogs correlated with those of owners. This may be due to different cognitive and affective states in dogs versus owners, which results from distinctive goals and focus of attention^58^ during the tasks. In both tasks, the dogs were possibly most interested in getting treats whether by following the owner’s commands or sniffing the mat, whereas the owners were concentrated on giving commands to the dogs during Training and mainly monitoring the sufficiency of treats during Sniffing. Treat eating in dogs but not in owners may have also resulted in different ANS activation and the respective arousal levels. Generally, eating activates parasympathetic nervous system and increases HRV^17^; however, here the dogs showed decreased HRV during Sniffing regardless of low activity levels. Similar results were found in our previous study^23^ where manipulating Kong, a toy filled with food, decreased the HRV of the dogs. Thus, we argue that eating tasty food invoked positive excitement (i.e. high arousal state) in dogs leading to low HRV values. Emotional states with high arousal are often linked to negative emotions^48^, such as fear and anger, but the decrease in HRV alone does not indicate whether the emotional state is negative or positive^59^. Our study together with previous research^23,60^ suggest that high arousal states with decreased HRV can also result from positive emotions.

### Factors explaining HRV in dogs and owners

One of our main questions was whether factors related to dog, owner, or their relationship could explain changes in HRV in the dogs and their owners. The *overall HRV of the dog* statistically significantly predicted the *owner’s overall HRV*; the RMSSD of the dog explained approximately 20% of the variance within the RMSSD of the owner. It is worth noting that the dog’s arousal level had a stronger connection with the physiology of the owner than the owner’s own characteristics (i.e. the weight, age, or temperament of the owner). Even the activity did not explain the owner’s arousal levels, which is somewhat surprising, considering that activity and HRV are closely linked to each other^19^. It is possible that the owners consciously intended to connect with their dogs throughout the study considering the transparent aim of this research, which may have improved observing of the dog and its arousal levels. Nevertheless, this result together with previous findings on hormonal synchronization^9,13,21^ establish the physiological connection between dog and owner during interaction.

The *overall HRV of dog* was predicted by *Dog’s height*, which had the strongest association with HRV; HRV values were greater in larger dogs. In dogs^36^ and other mammals^61^, larger body size generally leads to a lower heart rate and, consequently, to greater HRV. Interestingly, all the other significant predictors of *dog overall HRV* were related to the characteristics of the owner or the relationship between the dog and the owner instead of the dog itself. The *ownership duration* had a negative connection to HRV; the longer the dyad had been living together, the lower the *dog’s overall HRV* was during the study. In contrast, the *age of dog* did not influence the *dog’s overall HRV* despite the strong overlap between *the ownership duration* and the *age of dog,* demonstrating the specific impact of the total time lived together. As the positive correlation between *ownership duration* and *dog overall activity* indicates higher activity in dogs who had longer relationship history with their owners, dogs with longer time with the owner may have simply interacted more with the owner or exhibited increased exploratory behavior leading to greater activity^62^. Previously, dogs with a longer relationship history also showed lower HRV before and after gazing at the owner^63^, suggesting increased emotional arousal related to interaction with the owner over time. Furthermore, emotional contagion^33^ and owner-felt *Emotional closeness* with their dogs are stronger in dog–owner dyads with longer history together, addressing the role of time spent together and accumulated positive experiences in forming close emotional bonds. Taken together, the dogs who had a longer history with their owners may have more secure bonds and, as a result, greater confidence to explore the surroundings^8,62^. This increased explorative behavior is likely reflected in higher activity and lower HRV levels in these dogs.

In addition to the time that the dog and owner have lived together, the number of shared activities they practiced regularly *(Dog–owner Interaction)* also increased the arousal level of dogs during the study; the more the dog was considered as a family member and the more shared activities the owner reported in their everyday life, the lower the *overall HRV of the dog* was during the study. Since majority of our participants were active in dog sports, the dogs may have been expecting corresponding activities during our study, thus showing increased arousal manifested in lower HRV levels. Furthermore, the positive correlation between *Dog–owner Interaction* and *Emotional Closeness*, supported by previous findings^23,26^, indicates that owners who frequently spent time with their dogs form closer bonds with them than owners who spent less time with their dogs. Interestingly, frequent shared activities between the dog and the owner are also related to increased proximity-seeking behavior^64^ and higher morning cortisol levels^24^ in dogs. In our previous study, dogs with higher *Dog–owner Interaction* (MDORS-DOI) exhibited more arousal and attention-seeking behaviors and tended to have lower HRV^23^. Hence, the alternative explanation for lower HRV in dogs with more shared activities with the owner is that dogs prone to stress and anxiety seek more attention and require more devotion from the owner to overcome everyday challenges. Therefore, the owners probably pay more attention to the insecure dog, either intentionally or unintentionally, which in turn then enhances interaction between the dog and the owner and strengthens their emotional bond^9^. It is noteworthy that the MDORS subfactor *Dog–owner Interaction* has a relatively low Cr-α in this study, consistent with a previous study with a larger sample size of the Finnish dog owner population^26^. The results related to *Dog–owner Interaction* are, nevertheless, in line with previous findings.

Our results support previous observations, in which the owner’s temperament modify the physiology and behavior of the dog. The dogs of owners who had higher negative affectivity had higher overall HRV during the study, reflecting lower levels of arousal. Similarly, Sundman et al. found that dogs of more neurotic owners tend to have lower basal cortisol levels than dogs of less neurotic owners^13^. In both studies, the dogs represented herding and co-operative breeds specifically bred for close co-operation with humans, which may explain their sensitivity to the owner’s social cues^31^ and negative affectivity^26^. Interestingly, owners scoring higher in negative affectivity^26^ and neuroticism^65^ have stronger self-reported attachment and emotional closeness with the dog than owners scoring lower in these traits. One explanation to the appeasing influence of the owner’s negative affectivity and neuroticism on the dog is that these owners seem to experience greater social support from their dog and likewise provide more safety and emotional support for the dog^65^. Hence, closely bonded dog–owner dyads with a mutual tendency to require and provide emotional and social support might experience reduced stress and have greater stress resilience in novel situations. However, previous research related to the owner’s neuroticism and its effects on the dog–owner relationship is limited, and divergent results also exist^66,67^, indicating that multiple factors likely affect the outcome. This phenomenon is probably more multifaceted than generally understood and needs additional research.

To summarize, the results demonstrated a connection of physiological responses between dogs and their owners during interaction. Moreover, the owner’s temperament and the characteristics of the dog–owner relationship seem to affect the physiology and behavior of dogs belonging to co-operative breeds, which is consistent with previous findings^13,26,32^.

### Limitations and future direction

The current study was limited by the relatively small sample size. However, statistical analysis revealed moderate or strong effect sizes demonstrating the reliability of the findings. Our sample also consisted of mainly female dog owners, which is noteworthy as the owner’s gender may affect both the dog behavior^65^ and physiology^68,69^. Furthermore, the sample is likely biased towards active and committed dog owners, as most of the dyads practiced dog sports and were willing to participate in the study in their own free time without monetary compensation. Hence, it is plausible that dog–owner dyads with a more functional relationship than average are represented in this study.

Our dog sample was restricted to co-operative breed types such as herding dogs, as they were expected to show high responsiveness to humans^13,26^. Future research should also study ancient breeds in similar settings to determine whether the co-modulation of arousal and activity levels also exist between ancient breed dogs and their owners. In addition, considering the characteristics of the dog (e.g., personality or cognitive tests) and analyzing moment-by-moment HRV synchrony during the dog–owner interaction may elaborate the physiological co-modulation found between dogs and owners in more detail.

## Conclusions

The arousal and activity levels of co-operative breed dogs and their owners modulate each other during short-term interaction. The partly independent co-modulation of activity and HRV in dog–owner dyads suggest that emotional arousal is conveyed between dogs and their owners. We conclude that the physiological and emotional mechanisms involved in strengthening attachment bonds between humans also support the emotional relationship between humans and dogs.

## Supplementary Information

Supplementary Tables.

## Data Availability

The data supporting the findings of this study are not publicly available due to privacy and ethical restrictions but are available from the corresponding author on reasonable request. The metadata for this study can be found at the following link: 10.17011/jyx/dataset/96,732.
