# Supplementary material for: Behavioral and emotional co-modulation during dog–owner interaction measured by heart rate variability and activity
Source: Sci Rep. 2024 Oct 24;14:25201. doi: 10.1038/s41598-024-76831-x (PMC11502769; doi:10.1038/s41598-024-76831-x)
Supplement: Supplementary file 1 — Supplementary Tables. [file 41598_2024_76831_MOESM1_ESM.docx]

Supplementary material for: Behavioral and emotional co-modulation during dog-owner interaction measured by heart rate variability and activity

by Aija Koskela, Heini Törnqvist, Sanni Somppi, Katriina Tiira, Virpi-Liisa Kykyri, Laura Hänninen, Jan Kujala, Miho Nagasawa, Takefumi Kikusui & Miiamaaria V. Kujala

**Supplementary Table 1.** Breed and breed group of participant dogs.

| **Breed (Breed Group)** | **Dogs (n)** |
| --- | --- |
| Border Collie (FCI1)  Australian Shepherd (FCI1)  Rough Collie (FCI1)  Kelpie (FCI1)  Belgian Shepherd (FCI1)  Labrador Retriever (FCI8)  Mudi (FCI1)  Cocker Spaniel (FCI8)  German Shepherd (FCI1)  Bohemian Shepherd (FCI1)  Koolie (herding)  Nova Scotia Retriever (FCI8)  Portuguese Water dog (FCI8) | 5  4  3  3  3  3  2  2  1  1  1  1  1 |
|  |  |
|  |  |
|  |  |
|  |  |
| Total | 30 |

| **Supplementary Table 2.** The number of valid ECG segments achieved per each test phase and temporal alignment of the ECG segments of the owner and the dog. | | | | |
| --- | --- | --- | --- | --- |
|  | **Temporal alignment of the valid segments** | | |  |
| **Phase** | Exact  3 minutes^1^ | Exact  1-2 minutes^2^ | Partial  1-3 minutes^3^ | Invalid segments^4^ |
| Pre-Baseline | 23 | 1 |  | 1 o |
| Stroking | 23 |  | 2 |  |
| Training | 25 |  |  |  |
| Sniffing | 25 |  |  |  |
| Playing | 14 | 5 | 1 | 4 o, 1 d  1 (d) |
| Post-Baseline | 24 |  |  | 1 d |
| 1. Simultaneous ECG data achieved from both the dog and the owner for 3 minutes 2. Simultaneous ECG data achieved from both the dog and the owner for 1-2 minutes 3. Simultaneous ECG data achieved from the dog and owner for partially overlapping time intervals e.g. 2 minutes of valid data gathered from the owner and 3 minutes from the dog, from which one minute was recorded simultaneously from both parties 4. The number of invalid ECG segments, o = owner, d = dog | | | | |

**Supplementary Table 3.** Task-specific HRV and activity values of dogs and owners (n= 25). Values are given in mean ± SD (and median ± IQR).

| **Task** | **Dog RMSSD (ms)** | **Owner RMSSD (ms)** | **Dog activity \|v\|** | **Owner activity \|v\|** |
| --- | --- | --- | --- | --- |
| Pre-Baseline | 316.54 ± 254.24  (209.76 ± 392.13) | 31.55 ± 14.16  (28.36 ± 25.46) | 17.50 ± 16.52  (8.12 ± 19.07) | 6.73 ± 1.05  (6.24 ± 1.94) |
| Stroking | 163.40 ± 128.38  (129.99 ± 130.97) | 27.74 ± 13.72  (30.21 ± 23.01) | 24.49 ± 18.09  (17.99 ± 16.33) | 12.06 ± 4.37  (11.33 ± 5.56) |
| Training | 65.77 ± 39.35  (49.87 ± 56.66) | 19.55 ± 7.71  (19.74 ± 9.97) | 116.51 ± 60.74  (106.68 ± 77.50) | 23.44 ± 6.66  (24.51 ± 10.44) |
| Sniffing | 81.66 ± 58.22  (56.51 ± 54.08) | 35.23 ± 15.87  (38.60 ± 28.28) | 46.69 ± 15.79  (46.32 ± 19.95) | 9.65 ± 1.73  (8.94 ± 3.02) |
| Playing | 31.06 ± 17.21  (26.22 ± 19.36) | 17.66 ± 10.10  (14.62 ± 9.07) | 192.41 ± 73.27  (172.83 ± 131.77) | 57.92 ± 20.93  (59.63 ± 31.23) |
| Post-Baseline | 195.24 ± 205.80  (131.93 ± 205.75) | 30.58 ± 13.74  (27.31 ± 21.11) | 18.39 ± 14.30  (14.14 ± 23.35) | 6.76 ± 0.93  (6.55 ± 1.03) |

|v| = absolute value of accelerometer vector

**Supplementary Table 4.** The pairwise comparison of average HRV in dog owners during different tasks.

| **Sample 1 - Sample 2** | **Test statistic** | **Std. error** | **Std. test statistic** | **Sig.** | **Adj. sig.^a^** |
| --- | --- | --- | --- | --- | --- |
| Training – Playing | -0.300 | 0.592 | -0.507 | 0.612 | 1.000 |
| Training – Stroking | 2.000 | 0.592 | 3.381 | < 0.001 | 0.011 |
| Training – PostBaseline | -2.800 | 0.592 | -4.733 | < 0.001 | 0.000 |
| Training – PreBaseline | 3.000 | 0.592 | 5.071 | < 0.001 | 0.000 |
| Training – Sniffing | -3.000 | 0.592 | -5.071 | < 0.001 | 0.000 |
| Playing – Stroking | 1.700 | 0.592 | 2.874 | 0.004 | 0.061 |
| Playing – PostBaseline | -2.500 | 0.592 | -4.226 | < 0.001 | 0.000 |
| Playing – Sniffing | 2.700 | 0.592 | 4.564 | < 0.001 | 0.000 |
| Playing – PreBaseline | 2.700 | 0.592 | 4.564 | < 0.001 | 0.000 |
| Stroking – PostBaseline | -0.800 | 0.592 | -1.352 | 0.176 | 1.000 |
| Stroking – PreBaseline | 1.000 | 0.592 | 1.690 | 0.091 | 1.000 |
| Stroking – Sniffing | -1.000 | 0.592 | -1.690 | 0.091 | 1.000 |
| PostBaseline – PreBaseline | 0.200 | 0.592 | 0.338 | 0.735 | 1.000 |
| PostBaseline – Sniffing | 0.200 | 0.592 | 0.338 | 0.735 | 1.000 |
| PreBaseline – Sniffing | 0.000 | 0.592 | 0.000 | 1.000 | 1.000 |

Each row tests the null hypotheses that the Sample 1 and Sample 2 distributions are the same. Asymptotic significances (2-sided tests) are displayed. Total N = 20.

a. Significance values have been adjusted by the Bonferroni correction for multiple comparisons.

p < 0.05 indicates statistical significance

**Supplementary Table 5.** The pairwise comparison of average activity in dog owners during different tasks.

| **Sample 1 - Sample 2** | **Test statistic** | **Std. error** | **Std. test statistic** | **Sig.** | **Adj. sig.^a^** |
| --- | --- | --- | --- | --- | --- |
| PreBaseline – PostBaseline | -0.120 | 0.529 | -0.227 | 0.821 | 1.000 |
| PreBaseline – Sniffing | -1.560 | 0.529 | -2.948 | 0.003 | 0.048 |
| PreBaseline – Stroking | -2.120 | 0.529 | -4.006 | < 0.001 | 0.001 |
| PreBaseline – Training | -3.440 | 0.529 | -6.501 | < 0.001 | 0.000 |
| PreBaseline – Playing | -4.400 | 0.529 | -8.315 | 0.000 | 0.000 |
| PostBaseline – Sniffing | 1.440 | 0.529 | 2.721 | 0.007 | 0.098 |
| PostBaseline – Stroking | 2.000 | 0.529 | 3.780 | < 0.001 | 0.002 |
| PostBaseline – Training | 3.320 | 0.529 | 6.274 | < 0.001 | 0.000 |
| PostBaseline – Playing | 4.280 | 0.529 | 8.088 | < 0.001 | 0.000 |
| Sniffing – Stroking | 0.560 | 0.529 | 1.058 | 0.290 | 1.000 |
| Sniffing – Training | 1.880 | 0.529 | 3.553 | < 0.001 | 0.006 |
| Sniffing – Playing | -2.840 | 0.529 | -5.367 | < 0.001 | 0.000 |
| Stroking – Training | -1.320 | 0.529 | -2.495 | 0.013 | 0.189 |
| Stroking – Playing | -2.280 | 0.529 | -4.309 | < 0.001 | 0.000 |
| Training – Playing | -0.960 | 0.529 | -1.814 | 0.070 | 1.000 |

Each row tests the null hypotheses that the Sample 1 and Sample 2 distributions are the same. Asymptotic significances (2-sided tests) are displayed. Total N = 25.

a. Significance values have been adjusted by the Bonferroni correction for multiple comparisons.

p < 0.05 indicates statistical significance

**Supplementary Table 6.** The pairwise comparison of average HRV in dogs during different tasks.

| **Sample 1 - Sample 2** | **Test statistic** | **Std. error** | **Std. test statistic** | **Sig.** | **Adj. sig.^a^** |
| --- | --- | --- | --- | --- | --- |
| Playing – Training | 1.708 | 0.540 | 3.163 | 0.002 | 0.023 |
| Playing – Sniffing | 1.750 | 0.540 | 3.240 | 0.001 | 0.018 |
| Playing – PostBaseline | -2.583 | 0.540 | -4.783 | < 0.001 | 0.000 |
| Playing – Stroking | 3.042 | 0.540 | 5.632 | < 0.001 | 0.000 |
| Playing – PreBaseline | 4.167 | 0.540 | 7.715 | < 0.001 | 0.000 |
| Training – Sniffing | -0.042 | 0.540 | -0.077 | 0.939 | 1.000 |
| Training – PostBaseline | -0.875 | 0.540 | -1.620 | 0.105 | 1.000 |
| Training – Stroking | 1.333 | 0.540 | 2.469 | 0.014 | 0.203 |
| Training – PreBaseline | 2.458 | 0.540 | 4.552 | < 0.001 | 0.000 |
| Sniffing – PostBaseline | -0.833 | 0.540 | -1.543 | 0.123 | 1.000 |
| Sniffing – Stroking | 1.292 | 0.540 | 2.392 | 0.017 | 0.252 |
| Sniffing – PreBaseline | 2.417 | 0.540 | 4.475 | < 0.001 | 0.000 |
| PostBaseline – Stroking | 0.458 | 0.540 | 0.849 | 0.396 | 1.000 |
| PostBaseline – PreBaseline | 1.583 | 0.540 | 2.932 | 0.003 | 0.051 |
| Stroking – PreBaseline | 1.125 | 0.540 | 2.083 | 0.037 | 0.559 |

Each row tests the null hypotheses that the Sample 1 and Sample 2 distributions are the same. Asymptotic significances (2-sided tests) are displayed. Total N = 24.

a. Significance values have been adjusted by the Bonferroni correction for multiple comparisons.

p < 0.05 indicates statistical significance

**Supplementary Table 7.** The pairwise comparison of average activity in dogs during different tasks.

| **Sample 1 - Sample 2** | **Test statistic** | **Std. error** | **Std. test statistic** | **Sig.** | **Adj. sig.^a^** |
| --- | --- | --- | --- | --- | --- |
| PreBaseline – PostBaseline | -0.480 | 0.529 | -0.907 | 0.364 | 1.000 |
| PreBaseline – Stroking | -0.760 | 0.529 | -1.436 | 0.151 | 1.000 |
| PreBaseline – Sniffing | -2.040 | 0.529 | -3.855 | < 0.001 | 0.002 |
| PreBaseline – Training | -3.320 | 0.529 | -6.274 | < 0.001 | 0.000 |
| PreBaseline – Playing | -4.320 | 0.529 | -8.164 | < 0.001 | 0.000 |
| PostBaseline – Stroking | 0.280 | 0.529 | 0.529 | 0.597 | 1.000 |
| PostBaseline – Sniffing | 1.560 | 0.529 | 2.948 | 0.003 | 0.048 |
| PostBaseline – Training | 2.840 | 0.529 | 5.367 | < 0.001 | 0.000 |
| PostBaseline – Playing | 3.840 | 0.529 | 7.257 | < 0.001 | 0.000 |
| Stroking – Sniffing | -1.280 | 0.529 | -2.419 | 0.016 | 0.233 |
| Stroking – Training | -2.560 | 0.529 | -4.838 | < 0.001 | 0.000 |
| Stroking – Playing | -3.560 | 0.529 | -6.728 | < 0.001 | 0.000 |
| Sniffing – Training | 1.280 | 0.529 | 2.419 | 0.016 | 0.233 |
| Sniffing – Playing | -2.280 | 0.529 | -4.309 | < 0.001 | 0.000 |
| Training – Playing | -1.000 | 0.529 | -1.890 | 0.059 | 0.882 |

Each row tests the null hypotheses that the Sample 1 and Sample 2 distributions are the same. Asymptotic significances (2-sided tests) are displayed. Total N = 25.

a. Significance values have been adjusted by the Bonferroni correction for multiple comparisons.

p < 0.05 indicates statistical significance

**Supplementary Table 8.** Correlation coefficients (Spearman’s rho) between the study variables of dog–owner relationship, owner temperament, dog and owner demographics and overall HRV and activity. *p < 0.05; **p < 0.01; ***p < 0.001. All tests two-tailed.

|  | **MDORS** | | | **ATQR** | | | | **Owner characteristics** | | | | | **Dog characteristics** | | | | | |
| --- | --- | --- | --- | --- | --- | --- | --- | --- | --- | --- | --- | --- | --- | --- | --- | --- | --- | --- |
|  | **1 EC** | **2 PC** | **3 DOI** | **4 Ex** | **5 Sen** | **6 Neg** | **7 Eff** | **8 A** | **9 BMI** | **10 Ac** | **11 Hrv** | **12 Ow** | **13 A** | **14 He** | **15 Sex** | **16 Ac** | **17 Hrv** | **18 S** |
| Dog-Owner relationship (MDORS) | | | | | | | | | | | | | | | | | | |
| 1. Emotional closeness (EC) | 1 |  |  |  |  |  |  |  |  |  |  |  |  |  |  |  |  |  |
| 2. Perceived costs  (PC) | -0.261 | 1 |  |  |  |  |  |  |  |  |  |  |  |  |  |  |  |  |
| 3. Dog-Owner interaction (DOI) | 0.507^*^ | -0.303 | 1 |  |  |  |  |  |  |  |  |  |  |  |  |  |  |  |
| Owner temperament (ATQ-R) | | | | | | | | | | | | | | | | | | |
| 4. Extraversion (Ex) | 0.154 | -0.380 | 0.082 | 1 |  |  |  |  |  |  |  |  |  |  |  |  |  |  |
| 5. Orienting sensitivity (Sen) | 0.039 | -0.372 | 0.106 | 0.107 | 1 |  |  |  |  |  |  |  |  |  |  |  |  |  |
| 6. Negative affectivity (Neg) | 0.057 | 0.152 | 0.060 | 0.018 | 0.212 | 1 |  |  |  |  |  |  |  |  |  |  |  |  |
| 7. Effortful control (Eff) | -0.220 | -0.155 | 0.107 | -0.041 | -0.068 | -0.463^*^ | 1 |  |  |  |  |  |  |  |  |  |  |  |
| Owner characteristics | | | | | | | | | | | | | | | | | | |
| 8. Age (A) | -0.035 | -0.357 | -0.117 | 0.109 | 0.145 | 0.090 | 0.199 | 1 |  |  |  |  |  |  |  |  |  |  |
| 9. BMI | 0.239 | -0.134 | 0.052 | 0.375 | 0.176 | 0.056 | -0.099 | 0.146 | 1 |  |  |  |  |  |  |  |  |  |
| 10. Activity of owner (Ac) | 0.013 | 0.117 | 0.091 | -0.088 | -0.300 | -0.329 | 0.117 | -0.440^*^ | -0.214 | 1 |  |  |  |  |  |  |  |  |
| 11. HRV of owner | -0.367 | 0.120 | -0.151 | 0.212 | -0.272 | 0.134 | -0.046 | -0.092 | -0.127 | 0.058 | 1 |  |  |  |  |  |  |  |
| 12. Time of ownership (Ow) | 0.512^**^ | -0.028 | -0.045 | -0.180 | 0.026 | 0.017 | 0.145 | 0.098 | -0.136 | -0.096 | -0.249 | 1 |  |  |  |  |  |  |
| Dog characteristics | | | | | | | | | | | | | | | | | | |
| 13. Age (A) | 0.218 | 0.174 | -0.111 | -0.213 | 0.034 | 0.063 | 0.265 | 0.195 | -0.110 | -0.177 | -0.158 | 0.779^***^ | 1 |  |  |  |  |  |
| 14. Height (He) | 0.088 | -0.082 | 0.305 | 0.025 | 0.117 | 0.133 | -0.109 | -0.153 | -0.062 | 0.333 | 0.352 | -0.213 | -0.230 | 1 |  |  |  |  |
| 15. Sex | 0.162 | -0.145 | 0.123 | -0.228 | 0.095 | -0.467^*^ | 0.172 | 0.089 | -0.423^*^ | -0.033 | -0.344 | 0.370 | 0.330 | -0.228 | 1 |  |  |  |
| 16. Activity of dog (Ac) | 0.369 | 0.031 | 0.072 | 0.212 | -0.232 | -0.356 | 0.160 | -0.260 | 0.059 | 0.400^*^ | -0.215 | 0.397^*^ | 0.168 | -0.293 | 0.200 | 1 |  |  |
| 17. Hrv of dog | -0.297 | 0.001 | -0.065 | 0.176 | -0.170 | 0.244 | -0.272 | 0.096 | -0.079 | 0.018 | 0.527^**^ | -0.569^**^ | -0.315 | 0.568^**^ | -0.300 | -0.525^**^ | 1 |  |
| 18. Time of practicing dog sports (S) | 0.275 | 0.143 | -0.003 | -0.302 | 0.188 | 0.048 | -0.166 | -0.036 | -0.503^*^ | 0.025 | -0.372 | 0.530^**^ | 0.272 | -0.052 | 0.524^**^ | 0.160 | -0.369 | 1 |
